# A randomised controlled trial of a preconceptional dietary intervention in women undergoing IVF treatment (PREPARE trial)

**DOI:** 10.1186/1472-6874-14-130

**Published:** 2014-11-18

**Authors:** Alexandra J Kermack, Philip C Calder, Franchesca D Houghton, Keith M Godfrey, Nicholas S Macklon

**Affiliations:** Human Development and Health Academic Unit, Faculty of Medicine, University of Southampton, Southampton, SO16 6YD UK; Department of Obstetrics & Gynaecology, Princess Anne Hospital, Room F86, Level F, Coxford Road, Southampton, SO16 5YA UK; National Institute for Health Research Southampton Biomedical Research Centre, University Hospital Southampton NHS Foundation Trust and University of Southampton, Southampton, SO16 6YD UK; MRC Lifecourse Epidemiology Unit, University of Southampton, Southampton University Hospitals NHS Trust, Southampton, UK

## Abstract

**Background:**

In vitro fertilisation (IVF) treatment provides an opportunity to study early developmental responses to periconceptional dietary interventions. Retrospective studies have suggested links between preconception diet and fertility, and more recently, a "Mediterranean" diet has been reported to increase pregnancy rates by up to 40%. In addition, a prospective study examining increased intake of omega-3 polyunsaturated fats demonstrated a quickened rate of embryo development after IVF. However, up to now, few prospective randomised controlled trials have investigated the impact of periconceptional dietary interventions on fertility outcomes.

**Methods and design:**

The study is a randomised controlled trial of a dietary intervention consisting of olive oil for cooking, an olive oil based spread, and a daily supplement drink enriched with Vitamin D (10 microgram daily) and marine omega-3 fatty acids (2 g daily) for 6 weeks preconception versus a control diet of sunflower seed oil for cooking, a sunflower oil based spread, and a daily supplement drink without added Vitamin D or marine omega-3 fatty acids. Couples undergoing IVF will be randomised to either the intervention or control group (55 in each arm). The primary endpoint is embryo developmental competency in vitro, measured by validated morphokinetic markers. Secondary outcomes will include the effect of the dietary intervention on the nutritional content of the intrauterine environment.

**Discussion:**

This approach will enable rigorous examination of the impact of the dietary intervention on early embryo development, together with the influence of the peri-implantation intra-uterine nutritional environment.

**Trial registration:**

ISRCTN50956936

## Background

Large prospective cohort studies have demonstrated the impact of female and male preconceptional nutritional status on fertility, perinatal and long term health of the offspring [1]. More recently, research has shown that variations in preconceptional diet may impact IVF outcomes. A ‘Mediterranean’ diet high in vegetable oils, fish, vegetables and legumes and low in carbohydrate-rich snacks was positively associated with red blood cell folate and vitamin B6 in blood and follicular fluid and with a 40% increase in the probability of pregnancy [2]. The findings of a subsequent case control study by another research group also suggested that fertility outcomes were improved in couples with a Mediterranean diet [3]. In a further prospective study of the association between dietary intake of polyunsaturated fatty acids, significant correlations were observed between the reported dietary intake of the omega-3 fatty acids (FAs) alpha-linolenic acid and docosahexaenoic acid (DHA) and embryo morphology after IVF [4]. Despite this, no large prospective randomised intervention studies have investigated the impact of optimising the periconceptional diet on fertility outcomes, perinatal or child health outcomes [5]. Moreover, it remains unclear by which mechanisms nutritional modifications act to impact embryo quality, implantation and subsequent periconceptional and perinatal development.

One reason for the lack of data from intervention studies derives from the challenges involved in encouraging and monitoring compliance to specific dietary regimens, particularly when the intervention is rigorous and/or of long duration. However, recent data, primarily from rodent studies demonstrate that dietary manipulations within a very short window around the time of implantation can have profound effects on early development [6]. Administration of a maternal low protein diet during the 4 days of mouse pre-implantation development, followed by a control diet thereafter resulted in clear adaptive responses such as altered blastocyst numbers, upregulation of endocytosis into the yolksac, and an increase in the rate of trophoblast invasion. While these adaptive responses were observed to protect growth, they appeared to come at the cost of hypertension and abnormal behaviour patterns in adulthood [7]. Another group has shown that administering a high fat diet in this short period results in altered zygote viability [8], while treating with an insulin sensitizer during this period normalises the effects of obesity on oocyte quality and developmental competence [9].

While extrapolation from rodents to humans brings uncertainties, this work suggests that a relatively brief dietary intervention in the 6 weeks prior to conception could have measureable effects on biomarkers of gamete and embryo health, the intra-uterine nutritional environment, and subsequent fetal and placental development, with implications for the long-term health of the offspring. Such a short period is likely to increase willingness to take part in a dietary intervention study and improve compliance during the intervention period. In addition, some lipid pools will have achieved maximal, or near-maximal, changes in eicosapentaenoic acid (EPA) and docosahexaenoic acid (DHA) content within 6 weeks [10].

Studying the impact of short-term interventions in the general population is complicated by the difficulty of accessing couples in this preconceptional phase, and the relatively low probability of subsequent conception within one or two months of completing the intervention. These challenges are markedly alleviated in a population attempting to conceive by means of assisted conception techniques. Couples reporting for fertility treatment are readily accessed in the periconceptional phase, and have on average a 30% chance of conceiving in their first cycle of treatment. It is therefore feasible to study the impact of a relatively short dietary intervention, which by virtue of its limited duration is more likely to be adhered to for the duration required.

A further advantage of this model is the unique access IVF treatment gives to human gametes, embryos and the peri-implantation uterine environment. IVF offers a window on how the complex biological processes in early human development are affected by nutritional status. At the time of oocyte retrieval in IVF, follicular fluid and blood can be sampled and analysed for nutritional biomarkers. Since the fate of the oocyte can be followed in terms of morphology, fertilisation and embryo development, direct correlations between the nutritional environment of the developing oocyte and spermatozoa and IVF outcomes can be ascertained, as our group and others have shown previously [11]. At the oocyte level, high fat diets in rodents have been shown to lead to lipid accumulation and endoplasmic reticulum (ER) stress [12], while follicular fluid folate levels have been shown to correlate to embryo quality [13].

Embryo quality can be assessed in terms of implantation potential on the basis of morphological and developmental criteria. The recent availability of incubators fitted with time lapse video monitoring technologies represents a step change in the ability to time precisely when key milestones of development are reached (‘morphokinetics’). This non-invasive means of interrogating early human development is enhanced by the generation of a video of each embryo allowing detailed analysis of development markers at a later date [14]. In addition, analysis of embryo conditioned medium provides further non-invasive means of assessing the impact of periconceptional diet on embryo metabolism [15].

Recent techniques developed by our group also allow us to analyse the impact of preconceptional diet on the intra-uterine environment. We have previously demonstrated the utility and practicality of aspirating endometrial secretions at the time of embryo transfer, which allows the study of the immediate intrauterine environment encountered by the embryo [16]. It is now clear that uterine secretions have a key role in providing nutritional support for the pre-implantation embryo and recently the role of female reproductive tract fluid composition on developmental origins of later health has been highlighted [17]. Current work is investigating the amino acid and glucose content of human endometrial fluid. Moreover, we have developed in-vitro models of embryo-endometrial interaction which provide the means to study the impact of nutritional interventions on embryo-endometrial signalling using donated cryo-preserved embryos [18–20].

These approaches will enable rigorous examination of the impact of the dietary intervention on early embryo development, and the peri-implantation intra-uterine nutritional environment. Should a significant positive impact of a dietary intervention on early development be demonstrated, this would have major implications for health policy and strengthen arguments for the provision of preconceptional nutritional advice to the general population. The data generated will inform new approaches to improving preconceptional nutrition, and provide the basis for a major prospective community based intervention study aimed at determining the effectiveness of relatively short, low cost and feasible dietary intervention on pregnancy outcomes and child health.

This study will be conducted in compliance with the protocol, GCP and the applicable regulatory requirements.

## Methods and design

### Study objective

Although retrospective studies have suggested a link between preconception diet and human fecundity, several questions still remain for example which nutrients are beneficial and how long they need to be taken prior to conception. This prospective, randomised, controlled trial (the PREPARE trial (PREconception dietary suPplements in Assisted REproduction)) aims to address the questions of the effect of a diet rich in marine omega-3 FAs and Vitamin D on a developing embryo and the intrauterine environment.

### Hypothesis

Our hypothesis is that a diet rich in marine omega-3 FAs and Vitamin D will be beneficial to a developing embryo and thus improve morphokinetic markers used to predict embryo viability and implantation. Further to this, it is thought that this diet will affect the nutritional content of the intrauterine environment and alter the endometrial tissue immune cell populations.

### Study population and recruitment

Women participating in the PREPARE trial will be between the ages of 18 and 41 years, with a body mass index (BMI) between 20 and 32 kg/m^2^, and undergoing their first or second cycle of IVF or in vitro fertilisation – intracytoplasmic sperm injection (IVF-ICSI). Their partners must be using their own sperm and be prepared to provide a fresh sample prior to the dietary intervention and on the day of oocyte retrieval (they should not be using frozen sperm or sperm obtained by surgical retrieval). Additional inclusion criteria include an anti-műllerian hormone (AMH) of greater than 7 pmol/l or an antral follicle count (AFC) of more than 10. Participants must be willing to provide written informed consent to participate in the study.

Exclusion criteria are: any medical contraindication to IVF or IVF-ICSI treatment or to a specific dietary intervention, previously diagnosed diabetes, taking prescribed medication or herbal remedies apart from simple painkillers, and eating oily fish (as defined by the UK Food Standards Agency) more than once a week.

**Figure 1:**
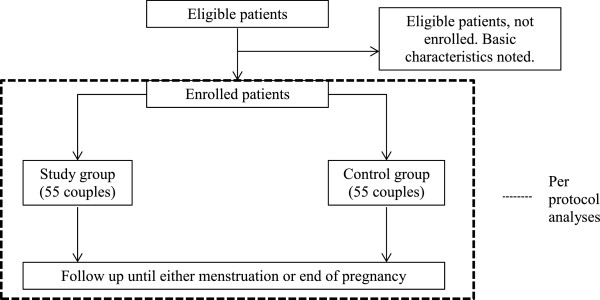
**Flowchart of eligible patients.**

Eligible patients will be informed about the PREPARE study during information evenings or their initial consultation with the medical team. A minimum reflection period of 3 days will be offered. Eligible patients who wish to participate will be randomised after providing written consent. If an eligible patient declines participation, basic characteristics will be obtained to identify any selection bias (Figure 1).

### Study design

The PREPARE trial is a single centre, randomised controlled trial, powered to demonstrate a difference in mean duration of the second cell cycle (CC2) of embryos generated by IVF after exposure of the couple to the intervention versus control. Both the male and female in the intervention group will receive a six week diet of olive oil for cooking, an olive oil based spread, and a daily supplement drink enriched with Vitamin D (10 micrograms) and the marine omega-3 FAs EPA (1 g) and DHA (1 g daily) versus the control diet of sunflower seed oil for cooking, a sunflower oil based spread, and a daily supplement drink without vitamin D, EPA or DHA.

Following inclusion into the study but prior to randomisation, consenting participants will be invited to complete a preconception questionnaire and the short Southampton Food Frequency Questionnaire (FFQ) in order to characterise their lifestyle and diet prior to entry into the study [21]. At this time, samples of blood, uterine fluid and endometrium will be obtained from the women in order to provide baseline data of the nutritional and immune cell content prior to the intervention. The male participants will be asked to provide a semen sample which will be analysed for concentration, motility and morphology according to the WHO criteria [22].

The women embarking on the study will undergo ovarian stimulation according to our standard protocol, provided no abnormality is seen on their baseline scan on day 2 of their cycle. Oocyte retrieval will be performed 36 hours following the single dose of human chorionic gonadotrophin (hCG), which promotes oocyte maturation. At the point of oocyte retrieval, further blood will be taken from the female participants and the follicular fluid and cumulus cells that are routinely removed, during IVF-ICSI, from the oocyte prior to insemination will be snap frozen. A further semen sample from the males will be analysed according to the WHO criteria prior to insemination.

The embryos will be cultured in Vitrolife IVF media in a validated time lapse incubator (Embryoscope, Unisense, FertiliTech, Denmark) in 5% CO2, 5%O2 and at 37°C. During the incubation, twenty one plane focal images will be generated every hour and analysed according to morphological and morphokinetic markers [23]. For women in whom embryo transfer is possible, uterine fluid aspiration will be carried out prior to the procedure. In those not undergoing embryo transfers, an endometrial biopsy will also be performed.

**Figure 2:**
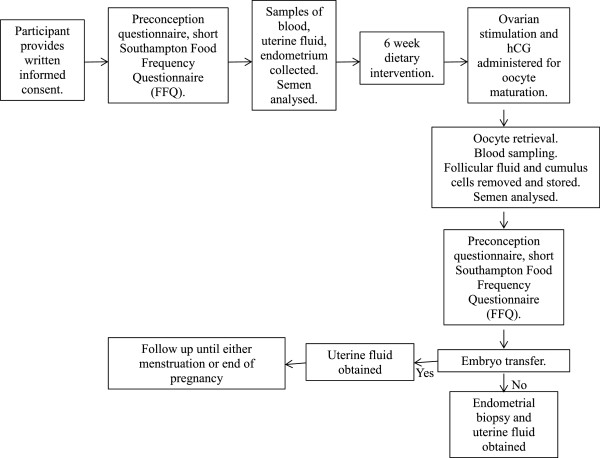
**Flow chart of study design.**

Compliance with the diet will be monitored by a daily diary completed at home by the couple and by counting the empty cartons when they are returned to the Fertility Unit (Figure 2).

A recent systematic review of current literature and trial registers identified no other randomised controlled trials (RCTs) addressing this subject.

### Primary and secondary endpoints

The primary endpoint is the mean period in hours for the cleaved embryos from participants in each arm of the study to develop from the 2 cell to the 3 cell stage (CC2). Secondary endpoints include using the Embryoscope to examine embryo quality, including S2 (synchrony during second set of cleavage divisions), T5 (time between fertilisation and the five cell embryo formation) [14, 24], CC3 (duration of the third cell cycle) [24], and the appearance and disappearance of the pronuclei [25]. Nutritional markers, including the concentration of fatty acids, Vitamins D, B12, and B6, folate, prostaglandin E2 (PGE2) and prostaglandin F2α (PGF2α) will be measured in blood, endometrial fluid and follicular fluid pre and post intervention. Changes in endometrial tissue immune cell populations will also be examined. In male participants, changes in semen quality pre and post dietary intervention will be recorded according to standard parameters.

Implantation rates and pregnancy outcomes will be recorded and analysed. These include antenatal ultrasound markers; such as crown-rump length at 7 weeks and 12 weeks and fetal head and abdominal circumference, femur length and cross sectional metaphyseal area and placental volume at 20 weeks gestation. Perinatal measurements including birthweight and length and placenta dimensions and weight will also be recorded. Participants will be invited to bring their neonate to undergo a Dual energy X-ray Absorptiometry (DXA) to measure bone mineral content and density soon after birth.

### Participating hospitals

This is a single centre study that will be conducted at the Complete Fertility Centre, a tertiary unit within the University Hospital Southampton NHS Foundation Trust.

### Randomisation

After collection of baseline data in the cycle prior to the intervention commencing, participating couples will be randomised to one or other of the study arms. The research team will be blinded to the randomisation. Stratification will be performed at randomisation for planned mode of fertilisation: in vitro fertilisation or intra-cytoplasmic sperm injection.

### Data collection

Data collection will be performed using a case report form (CRF). Data relating to clinical treatment outcomes, including embryo quality measurements will be acquired from the Fertility Unit’s electronic data collection system. Questionnaire responses will be inputted electronically into the CRF. Additional data relating to laboratory assays will be obtained directly from the laboratory’s database or the respective external laboratories and entered into the CRF. This minimises the risk of transcription errors; however, all data will be verified and cleansed prior to analysis.

### Statistical analysis

#### Sample size and power considerations

In a previous retrospective study from our group, a diet rich in the omega-3 FAs was shown to be associated with improved embryo morphology when assessed on day 3 post fertilisation. Using a published scoring system between 1 (highest quality) and 5 (lowest quality), embryos generated in women with a daily mean total intake of omega-3 FAs in the 4 weeks prior to IVF greater than 1.7 g scored a mean of 0.6 (SE 0.26) points higher than those embryos derived from a woman reporting an intake of <1 g per day. Recently, morphokinetic analysis of human embryos has shown the duration of key development phases to be correlated to standard conventional morphological criteria, but more highly predictive of implantation potential [14, 26]. One of the most sensitive predictors of implantation has been shown to be CC2. Embryos with a CC2 shorter than 11.9 hours (pooled SD 2.25 hours, but not normally distributed) have been reported to have an implantation rate of 35% compared to 28% when this key developmental step took longer than 11.9 hours. Moreover, in a study correlating morphokinetic parameters with static morphology scoring, a CC2 less than 11.9 hours was shown to correlate with an overall increase in embryo score of 0.5 points on a 5 point scale [14]. This is similar in magnitude to the impact on embryo morphology reported to be achieved by exposure to a high omega 3 FA diet as described above [4].

The primary end point of this study is therefore the difference in mean CC2 score of embryos generated by IVF after exposure of the couple to the intervention versus control diet. In order to detect a minimum absolute difference of 12% (1.4 hours) in mean CC2, or effect size of 0.670, with power ≥80% at p <0.05 a non-parametric comparison (Wilcoxon test) indicates a requirement of 46 couples per group in the analysis.

To allow for drop outs and failure to produce sufficient viable embryos, a further 20% will be recruited. This requires the randomisation of a minimum of 55 couples per group (110 in total).

### Data analysis

In order to adjust for pre-defined confounders such as age, AMH level, ovarian stimulation method, fertilisation method and the number of oocytes obtained, univariate and multivariate analyses will be performed. This will enable the independent contribution of the dietary factor to the primary and secondary outcome measures to be quantified.

Differences in embryo quality parameters will be tested by Wilcoxon testing, and differences in blood and endometrial fluid levels of the stated secondary outcome parameters will be tested by the Mann Whitney U test.

### Ethics

This study is designed using the guidelines for good clinical practice (GCP) as well as the Declaration of Helsinki 1964 as revised and recognised by governing laws and EU Directives. Full ethical approval (13/SC/0544) was granted from South Central (Oxford A) Research Ethics Committee (NRES) via the Integrated Research Application System (IRAS). In accordance with the GCP guidelines, written informed consent prior to randomisation will be mandatory.

## Discussion

With this study we aim to clarify whether a diet rich in Vitamin D and marine omega-3 FAs significantly improves embryo development by improving morphokinetic markers of embryo quality. Moreover, we will identify how a change in diet for six weeks alters the nutritional and immune environment of the developing oocyte, sperm and implanting embryo.

The decision to use morphokinetic markers of embryo quality as the primary endpoint rather than number of live births was taken for a number of reasons. Primarily, clinical pregnancy rate reflects the success of IVF treatment in subfertility and while dietary intervention may contribute to this, so do a number of other factors including the number of oocytes obtained following ovarian stimulation with exogenous gonadotrophins. Therefore, if embryo quality is shown to improve with the dietary intervention, it is more applicable to the general population and is likely to be of benefit to all trying to conceive and to their offspring. Secondly, the number needed to recruit in a trial looking at live birth rates would be far greater and therefore outside the ability of this study.

It is hoped that the short duration and simplicity of the intervention will improve both willingness to participate in the study and compliance. To our knowledge, this will be the first prospective randomised controlled trial in humans that examine a dietary intervention and embryo quality. We consider this study to be achievable within the planned time frame of 3 years.

## Abbreviations

AFC: Antral follicle count
AMH: Anti-mullerian hormone
BMI: Body mass index
CC2: Duration of the second cell cycle
CC3: Duration of the third cell cycle
CRF: Case report form
DHA: Docosahexaenoic acid
DXA: Dual energy X-ray absorptiometry
EPA: Eicosapentaenoic acid
ER: Endoplasmic reticulum
FAs: Fatty acids
GCP: Good clinical practice
hCG: Human chorionic gonadotrophin
IRAS: Integrated research application system
IVF: In vitro fertilisation
IVF-ICSI: In vitro fertilisation – intracytoplasmic sperm injection
NRES: Research ethics committee
PGE2: Prostaglandin E2
PGF2α: Prostaglandin F2α
RCT: Randomised controlled trial
S2: Synchrony during the second set of cleavage divisions
T5: Time between fertilisation and the five cell embryo formation
WHO: World Health Organisation.
